# Therapeutic Approach in Pigmented Purpuric Dermatoses—A Scoping Review

**DOI:** 10.3390/ijms25052644

**Published:** 2024-02-24

**Authors:** Agnieszka Kimak, Agnieszka Żebrowska

**Affiliations:** Department of Dermatology and Venereology, Medical University of Lodz, Hallera 1, 90-647 Lodz, Poland; agnieszka.zebrowska@umed.lodz.pl

**Keywords:** Schamberg disease, pigmented purpuric dermatoses, capillaritis, treatment

## Abstract

Pigmented purpuric dermatoses (PPD) encompass a group of chronic skin conditions characterized by the presence of petechiae, purpura, and pigmentation changes. While generally benign, these dermatoses can be persistent and aesthetically bothersome. Key clinical features include red to brownish patches with a distinctive “cayenne pepper” appearance, predominantly localized on the lower extremities, particularly the shins. Subtypes include Schamberg disease, Majocchi’s disease, Gougerot–Blum disease, Ducas and Kapetanakis pigmented purpura, and lichen aureus. Diagnosis relies primarily on clinical evaluation of skin lesions, with biopsy as a confirmatory tool. Although the exact cause of PPD remains unclear, capillary fragility and red blood cell extravasation are implicated. Treatment strategies for PPD aim to alleviate symptoms, considering the generally benign and chronic nature of the condition. As there is no standardized treatment, various methods with varying efficacy are employed. After searching SCOPUS and PubMed databases, we assessed 42 original articles to present current knowledge regarding therapy of PPD. This review will compare treatment approaches specifically in Schamberg disease and other manifestations of pigmented purpuric dermatoses.

## 1. Introduction

Pigmented purpuric dermatoses (PPD) encompass a cluster of skin disorders marked by petechial hemorrhage resulting from capillaritis. PPD are considered relatively uncommon but can affect individuals of all ages, including children [1,2]. Schamberg disease (SD) is the predominant variant observed in both adults and children [3].

### 1.1. Pathogenesis

**Figure 1:**
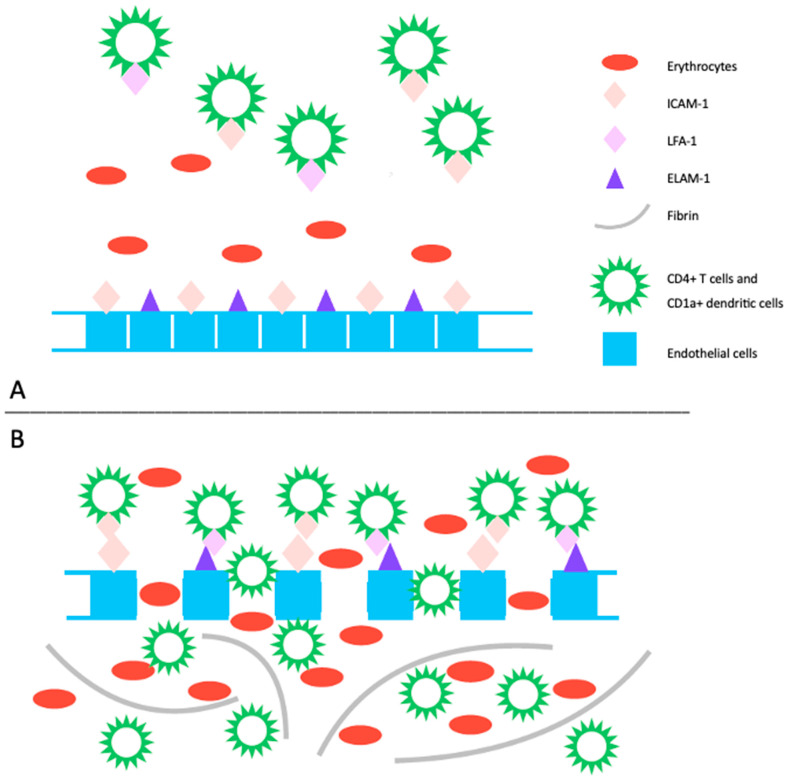
(**A**) The pathogenic mechanism of pigmented purpuric dermatoses involves a spontaneous or triggered immunologic event that induces an upregulation of cell adhesion molecules. This includes increased expression of ICAM-1 on endothelial cells, CD4+ T cells, and CD1+ dendritic cells, as well as ECAM-1 on endothelial cells, and LFA-1 on CD4+ T cells and CD1+ dendritic cells. (**B**) This heightened expression promotes the migration and adhesion of CD4+ T cells and CD1a+ dendritic cells, leading to increased endothelial permeability and disruption of dermal capillaries, resulting in extravasation of erythrocytes. Subsequently, this process triggers an immune response primarily mediated by T lymphocytes, leading to perivascular inflammatory infiltrate. Additionally, activated T-cells impact the plasminogen pathway, contributing to intraperivascular fibrin deposition. LFA-1: leukocyte function adhesion 1, ELAM-1: endothelial leukocyte adhesion molecule 1, ICAM-1: intercellular adhesion molecule 1.

The pathogenesis of PPD is not completely understood, and it may vary among the different subtypes. However, common features include inflammation and hemorrhage of superficial papillary dermal vessels, primarily capillaries. Studies suggest an immunologic event involving cell adhesion molecules (CAM) like leukocyte function adhesion 1 (LFA-1), endothelial leukocyte adhesion molecule 1 (ELAM-1), and intercellular adhesion molecule 1 (ICAM-1), indicating a cell-mediated hypersensitivity reaction [4]. The perivascular inflammatory infiltrate includes CD4+ T cells and CD1a+ dendritic cells. Cytokines produced by leukocytes may induce CAM expression, affecting fibrinolytic activity and causing intravascular fibrin deposition observed in PPD [4,5]. Capillary dilation and fragility, possibly attributed to altered function of blood vessel structure cells like fibroblasts and endothelial cells, may lead to red blood cell leakage, triggering an immune response (Figure 1). Another emerging theory suggests PPD may represent an epitheliotropic T-cell alteration, supported by observations of epidermotropism or a monoclonal pattern in the inflammatory infiltrate [6]. Progression to mycosis fungoides has been reported, emphasizing the need for integrated clinical, molecular, and histopathologic assessments for accurate diagnosis and management decisions [7,8].

### 1.2. Histopathology

**Figure 2:**
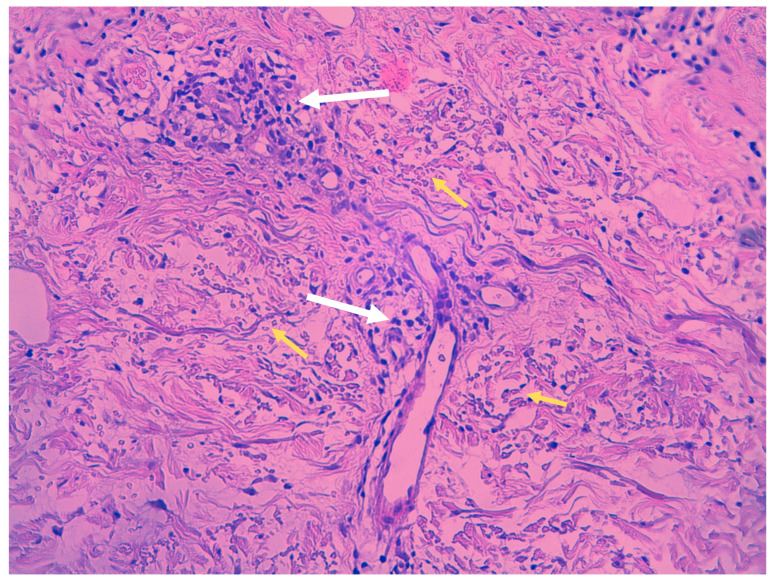
Histopathologic features of Schamberg disease. Lymphocytic infiltrate mainly involving capillaries (white arrows) and extravasated red blood cells (yellow arrows) (hematoxylin-eosin stain, 40× magnification).

Despite clinical variations, all PPD share common histopathological features, including blood vessels dilation, swelling of the endothelial cells, perivascular lymphocytic infiltration, erythrocytes extravasation, and hemosiderin-filled macrophages (Figure 2). Those features are common in all PPD, with distinctions in intensity noted in certain subtypes. Hemosiderin deposits in the superficial dermis, discerned through Perl’s and Fontana-Masson staining, differentiate PPD from stasis dermatitis [9,10]. The infiltrate predominantly consists of CD4+ lymphocytes, occasional CD1a+ dendritic cells, and macrophages [11]. Plasma cells and neutrophils may be present, with the latter observed in itching purpura lesions, such as eczematoid purpura of Doucas and Kapetanakis [11]. Mild epidermal spongiosis and lymphocytic exocytosis are common in all variants except lichen aureus, which typically shows a band-like infiltrate separated from the epidermis by a thin rim of uninvolved collagen (Grenz area) [12]. This infiltrate is also seen in pigmented purpuric lichenoid dermatosis of Gougerot and Blum [13]. A rare variant of granulomatous PPD with a superimposed hemorrhagic granulomatous infiltrate has been described as well [14]. Positive direct immunofluorescence may reveal the deposition of fibrinogen, IgM, and/or C3 in superficial dermal vessels [9]. In addition to confirming the diagnosis of pigmented purpuric eruption, a skin biopsy serves as a valuable tool for ruling out cutaneous T-cell lymphoma, especially in its early stages, as it can mimic PPD both clinically and histologically [8]. Besides a skin biopsy, it is advisable to undergo a blood test to exclude autoimmune disorders, thrombocytopenia, clotting abnormalities, and chronic infections, including HBV and HCV hepatitis [3].

### 1.3. Dermoscopy

**Figure 3:**
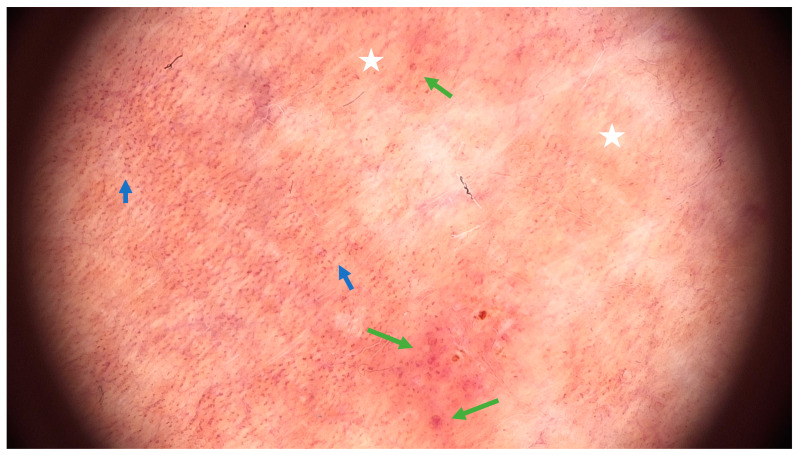
Dermoscopic features of Schamberg disease. Coppery-red background (white asterix), red dots (blue arrow) and globules (green arrow) (FotoFinder, FotoFinder Systems GmbH, Bad Birnbach, Germany, 20× magnification).

Examining pigmented purpuric dermatoses (PPD) through dermoscopy unveils unique characteristics facilitating their diagnosis. In PPD dermoscopy, a base coloration ranging from reddish-brown to orange is evident, mirroring the underlying hemosiderin deposition. Red dots and globules, corresponding to dilated capillaries and extravasated red blood cells, are commonly observed, contributing to the characteristic purpuric appearance. Moreover, a network pattern in shades of yellowish-brown, reminiscent of branched vessels, may be discerned, underscoring the vascular nature inherent to these dermatoses [15]. Less common features include brown or red circles, grey dots, serpentine or thick linear vessels, as well as rosette structures [16] (Figure 3).

### 1.4. Differential Diagnosis 

The differential diagnosis for pigmented purpuric dermatoses (PPD) includes several skin conditions that may share similar clinical or histopathological features, particularly involving the lower extremities. Prominent entities include drug hypersensitivity reactions, with potential involvement of specific medications like carbamazepine and nitroglycerine. Purpuric contact dermatitis to clothing is characterized by lesions confined to areas in contact with clothing, accompanied by intense itching, often associated with materials like wool and coloring agents. Venous stasis purpura manifests with signs of chronic venous insufficiency, including swelling, varicose veins, and venous ulcers. Other considerations encompass conditions such as purpura due to thrombocytopenia, senile purpura in the elderly, purpuric exanthema attributed to viral infections, leukocytoclastic vasculitis, IgA vasculitis (Schönlein-Henoch purpura) observed commonly in pediatric cases, Kaposi sarcoma affecting elderly or immunosuppressed individuals, and purpuric mycosis fungoides [3,10].

### 1.5. PPD Variants

**Figure 4:**
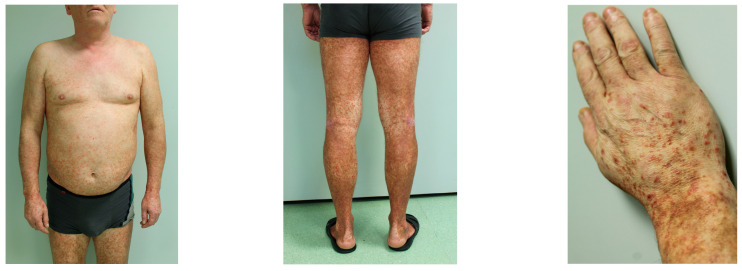
Clinical manifestation of Schamberg disease. Typical skin lesions include orange-red macules more prominent and coalescent on lower extremities.

Five classic subtypes of PPD are summarized in Table 1 and include: progressive PPD (Schamberg disease), pigmented purpuric lichenoid dermatosis of Gougerot and Blum, lichen aureus, purpura annularis telangiectodes (Majocchi disease), and eczematoid purpura of Doucas and Kapetanakis (Table 1, Figure 4) [3,17]. Other less common variants include Hersch and Schwayder’s linear unilateral form of PPD (distinct from linear variants of Schamberg disease and lichen aureus) [18], quadratic form described by Higgins and Cox [19], itching purpura of Loewenthal (sometimes considered as a symptomatic variant of SD affecting adults) [20], granulomatous PPD [14], and familial PPD [21]. Furthermore, a transient, estrogen-dependent variant of PPD, called angioma serpiginosum, has been documented in the literature [22].

## 2. Methods

**Figure 5:**
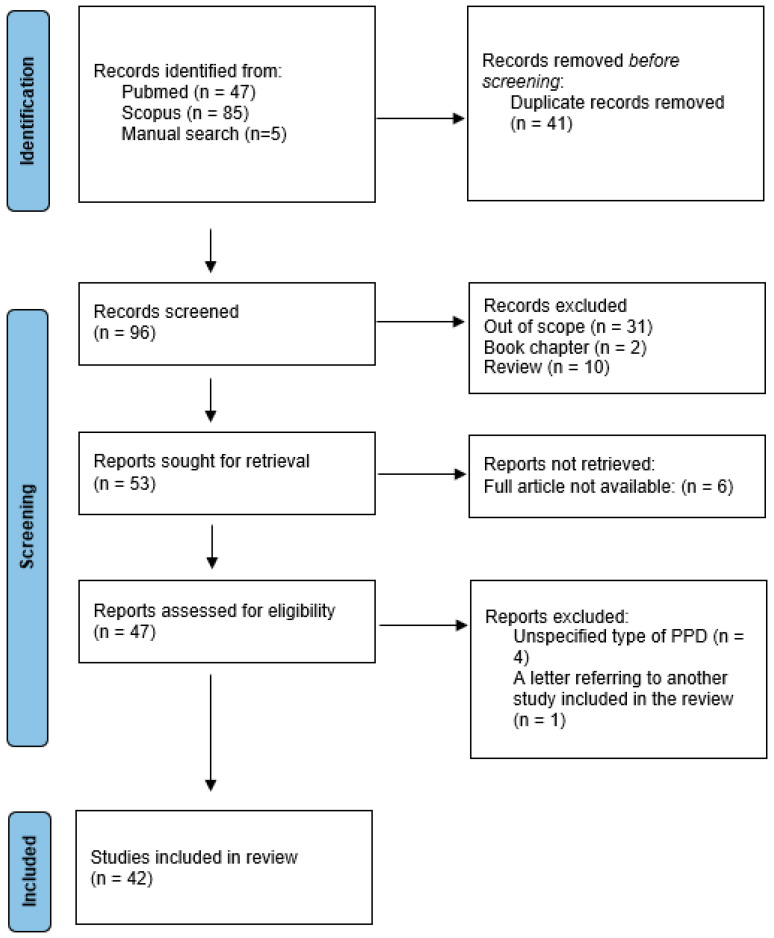
Flowchart on the selection and evaluation of scientific articles.

A research study on Schamberg’s disease was conducted on 21 December 2023 by AK. The search included terms “Schamberg *”, “purpura pigmentosa progressive”, “progressive pigment *”, and “treatment” in the articles written in English or Polish in the SCOPUS and PubMed databases; in total, 132 potentially eligible articles were detected. Additional relevant publications were obtained by reviewing the references from the chosen articles. After the removal of duplicates and irrelevant articles based on the titles, abstracts, and full text articles, 42 original articles were included in this review (Figure 5).

## 3. Triggering Factors of PPD

Although the exact cause of Schamberg disease is not well understood, certain medications have been implicated in the development or exacerbation of the disease, including amlodipine [23,24], raloxifene [25], dupilumab [26], and TNF-alfa inhibitors [27]. Alcohol [28], physical exercise [29], and odontogenic infection [30] have been attributed a possible pathogenic role in SD as well. Other drugs that have been linked to PPD eruptions include analgesics (aspirin, acetaminophen, nonsteroidal anti-inflammatory drugs (NSAIDs), zomepirac sodium), antibiotics, cardiovascular drugs, sedatives, antihyperglycemics, statins, retinoids, stimulants, antivirals, and chemotherapeutics [31]. Usually, discontinuation of the implicated drug leads to the resolution of skin lesions within a few weeks to months. Moreover, vitamins, supplements, as well as oral microbiota [32], COVID-19 vaccine [33], energy drinks, and hepatitis B infection have been described in association with PPD [31,34]. It is hypothesized that the drug or substance could act as a hapten, leading to antigen formation. The antigen–antibody interaction may stimulate an immune system-mediated injury near the capillary endothelium, resulting in vessel wall damage, and thus erythrocyte leakage [31,35]. It is important to note that many of these associations are based on case reports, and causality may not be firmly established.

## 4. Pigmented Purpuric Dermatoses—Treatment Options

The management of pigmented purpuric dermatoses poses challenges, as existing treatment options often yield unsatisfactory results. Various approaches, including the application of topical agents, oral medications, phototherapy, and laser treatment, have been employed, albeit with limited success. In the following section, we will delve into the treatment options available for addressing conditions like Schamberg disease and other forms of PPD.

### 4.1. Topical Treatment

Topical corticosteroids, commonly employed in the treatment of PPD, including Schamberg disease [2,30,36,37,38,39,40,41,42], often yield unsatisfactory effects [2,36,39,40,41,43]. Oral corticosteroids are similarly ineffective due to a high recurrence rate after dose tapering [44,45]. Although a combination of oral and topical steroids may yield better results, recurrences after treatment cessation are frequently observed [37,46]. Combinations of topical steroids with other agents, such as oral vitamin C [47] or vitamin C and rutoside [48] have been proven ineffective. Partial temporary responses were reported in patients undergoing a combined treatment of topical steroids with oral antihistaminic drugs [49] and antihistaminic drugs, vitamin C, rutoside, diosmin, and hesperidin combination [49].

Typically, topical steroids with various potency are more frequently employed in the pediatric population for PPD, serving as an alternative to oral treatment. However, prolonged use may be necessary to achieve results, necessitating consideration of potential side effects, particularly local skin atrophy and increased vascular fragility [43]. In cases of itching skin lesions, such as in purpuric lichenoid dermatitis of Gougerot–Blum, topical corticosteroids may be a viable option due to their effective and rapid improvement of pruritus [50].

Alternatively, topical calcineurin inhibitors—pimecrolimus and tacrolimus—could be considered, as they present a better side effect profile and have been proven effective in lichen aureus [51,52] and eczematoid purpura of Doucas and Kapetanakis [2], but not in pigmented purpuric lichenoid dermatosis of Gougerot and Blum [2]. Topical formulations containing vitamin K [2,42] and topical antibiotics [2,39] have proven ineffective in treating Schamberg disease and are not advisable for recommendation.

### 4.2. Pentoxifylline (PTX)

The effects of pentoxifylline (PTX) have been described in eight reviewed articles, and in total, the drug has been used in 187 patients [43,44,53,54,55,56,57,58]. Dosages ranging from 200 mg to 1200 mg per day were employed. Kano et al. utilized the lowest reported doses of 200–300 mg of pentoxifylline in three patients with SD. Notably, significant improvement was observed within a short period of 2 to 3 weeks, and the therapy was continued for up to 8 weeks. One patient experienced a recurrence after 4 months, which was successfully treated with another course of PTX [56]. In later studies, low doses of PTX (400 mg a day) were proven not be as effective [53,55,57,58].

Conversely, a daily dose of 1200 mg yielded a satisfactory effect in the majority of the patients, as highlighted in the reviewed articles [43,44,54]. In a comparative randomized trial, oral pentoxifylline was pitted against a topical steroid (betamethasone) [43]. Intriguingly, PTX demonstrated greater efficacy at 2 months, although this advantage diminished by the 6 month mark of treatment. Notably, there was no deterioration observed in the PTX group, while almost one in five patients in the steroid group experienced progression [43]. Taking into account this and the side effects profiles of both drugs, PTX appears to be superior to topical steroid treatment. Pentoxifylline has been also administered to a patient with acute viral hepatitis B; however, determining whether the improvement of skin lesions resulted from pentoxifylline administration or the clearance of the viral infection proves challenging [34].

Pentoxifylline is a xanthine derivative, predominantly employed for intermittent claudication associated with peripheral artery disease [59]. Presently, its applications extend to diverse conditions marked by inflammatory and vascular components. Its influence is mediated through varied mechanisms, with notable actions encompassing the inhibition and modulation of leukocytes, erythrocytes, platelets, and the vascular wall. Pentoxifylline, by exerting a non-selective inhibition of phosphodiesterase, particularly phosphodiesterase 4 (PDE-4), elevates intracellular cyclic adenosine monophosphate (cAMP) [60]. This results in the amplification of signaling pathways and downregulation of nuclear factor κB (NFκB), yielding various effects, including relaxation of vascular smooth muscle cells, enhancing blood flow; however, the precise mechanisms remain unclear [61,62].

**Figure 6:**
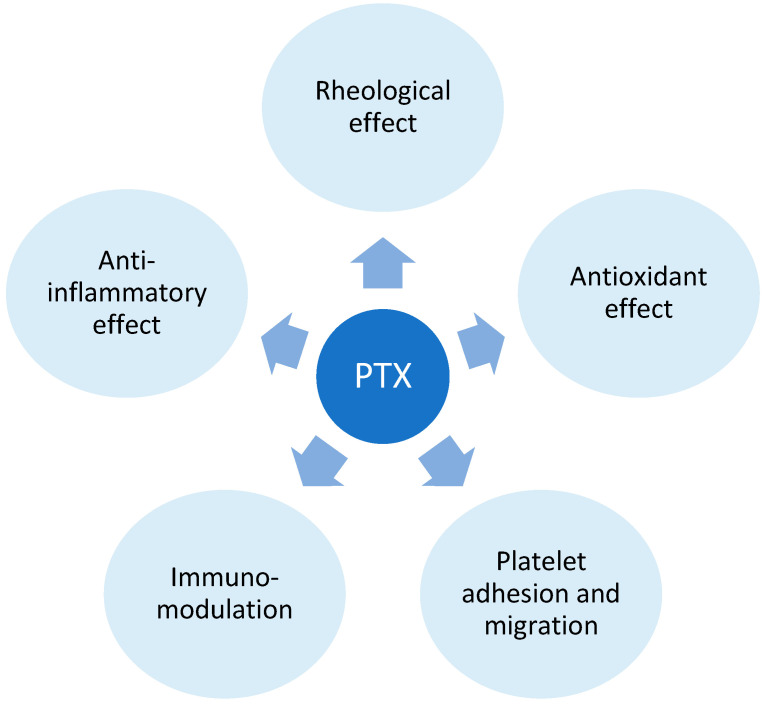
Pentoxifylline (PTX) mechanism of action. The mechanism of action of Pentoxifylline (PTX) involves its impact on blood viscosity, erythrocyte and platelet function, and the response of immune cells. These effects are primarily mediated by PTX’s influence on phosphodiesterase 4.

Additionally, cAMP plays a role in suppressing the transcription of pro-inflammatory cytokines such as TNF-alpha, interleukin (IL) 1, and IL-6 [63,64]. The anti-inflammatory impact involves a reduction in macrophage and lymphocyte infiltration, inhibition of monocyte chemoattractant protein (MCP-1), decreased expression of major histocompatibility complex class II (MHC II) antigens, and the inhibition of NF-κB activation [65,66]. PTX’s hemorheological effect improves red blood cell deformability, reduces blood viscosity, and diminishes the potential for platelet aggregation and blood clot formation. [60]. In addition to the immunomodulatory effects of PPE inhibition, PTX operates through adenosine-dependent pathways, curbing the activity of immune cells during acute inflammation [67]. The antioxidant impact of PTX is proposed to stem from the preservation of glutathione levels and mitochondrial viability, as well as by neutralizing free radicals and reactive oxygen species [68,69] (Figure 6).

Kano et al. conducted an immunohistochemical study, comparing dermal infiltration and the expression of keratinocytes and endothelium before and after treatment. Their findings revealed a decrease in infiltrate density, ICAM-1, and E-selectin expression, confirming the findings from previous in vitro and vivo studies on PTX [56].

### 4.3. Phototherapy

Among the screened articles, psoralen and UV-A (PUVA) photochemotherapy has demonstrated successful outcomes in patients with PPD as well [37,42,70,71]. The treatment typically involved fewer than 30 sessions, with cumulative doses ranging from 29 [71] to 200 J/cm^2^ [42]. PUVA has also shown effectiveness in conditions such as lichen aureus [72], Majocchi disease [73], pigmented purpuric lichenoid dermatosis of Gougerot and Blum [74] and unilateral linear capillaritis [75].

Narrowband UVB therapy (UVB-NB) has proven successful in the past for treating Schamberg disease [2,37,46,49,76,77,78,79]. The majority of cases demonstrated a positive response, with only a few instances of recurrence that were effectively addressed through retreatment [78]. Maintenance therapy of approximately nine sessions over 6 weeks could be advised for achieving long-term remission [78]. In certain cases, patients may necessitate extended maintenance therapy, as exemplified by a 33-year-old male who continued UV therapy once every two weeks to proactively prevent recurrences [46]. Many reports propose a potential utility of UVB-NB in various clinical presentations of PPD [78,80,81].

Phototherapy appears to have immunomodulatory and anti-inflammatory effects that contribute to its efficacy in managing PPD [82]. Furthermore, UVB light has been identified to trigger apoptosis in epidermal and dermal lymphocytes T within psoriatic lesions [83]. UVB-NB and PUVA therapies are cost-effective, generally well tolerated, and yield favorable therapeutic outcomes. Phototherapy is a viable consideration for treating PPD, particularly in cases involving extensive skin lesions that have shown resistance to previous treatments or in pediatric patients.

### 4.4. Vitamin C, Flavonoids and Calcium Dobesilate

Hemorheological agents (calcium dobesilate, bioflavonoids) and vitamin C have been employed for Schamberg disease management. In the examined studies involving a total of 37 patients, the combination of 500 mg of ascorbic acid twice daily and 50 mg of rutoside twice daily led to an improvement in skin lesions or complete remission [2,45,84]. In one case where the patient received ascorbic acid, rutoside, and topical steroids, no improvement was noted, although the article did not specify the dosage and duration of the treatment [48]. Another successful combination involved vitamin C and calcium pantothenate [85]. More intricate drug combinations, such as diosmin/hesperidin/Euphorbia prostata extract/calcium dobesilate [86] or antihistaminic drug/topical steroid/ascorbic acid/rutoside/hesperidin/diosmin [49], also resulted in remission. Regarding other forms of PPD, a male patient suffering from eczematoid purpura of Doucas and Kapetanakis [87] benefited from the standard treatment combination of 500 mg of ascorbic acid twice a day and 50 mg of the bioflavonoid rutoside twice a day. Similarly, three patients with an unspecified variant of PPD also responded positively to the same treatment [88].

**Figure 7:**
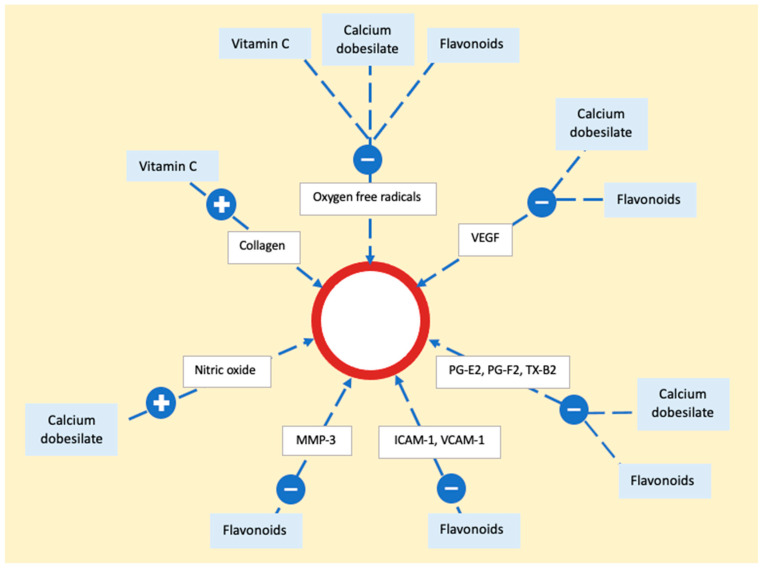
The mechanism of action of vitamin C, calcium dobesilate, and flavonoids on the epithelium. The process involves neutralization of oxygen free radicals by all three substances. Flavonoids and calcium dobesilate play a role in reducing the activity of vascular endothelial growth factor, prostaglandins, and thromboxane. Additionally, flavonoids contribute to a decrease in adhesion molecules and metalloproteinase, while calcium dobesilate stimulates nitric oxide synthesis. Vascular endothelial growth factor (VEGF), prostaglandin E2 (PG-E2), prostaglandin F2 (PG-F2), thromboxane B2 (TX-B2), metalloproteinase 3 (MMP-3), intercellular adhesion molecule 1 (ICAM-1), vascular cell adhesion molecule 1 (VCAM-1).

Vitamin C (ascorbic acid) functions as an electron donor, and serving as a reducing agent, it acts as a cofactor for numerous enzymes. In the context of treating PPD, its role lies in acting as a cofactor for hydroxylases that are integral to collagen synthesis [89]. By promoting collagen production, it enhances the strength of blood vessel walls and reduces endothelial permeability. Additionally, vitamin C diminishes local inflammation by reducing reactive oxygen species [90]. Alongside that, calcium dobesilate and bioflavonoids act as potent antioxidants, inhibiting the production of oxygen free radicals and lipid peroxidation, resulting in decreased synthesis of prostaglandins E2 or F2 and thromboxane B2 [82]. They also downregulate vascular endothelial growth factor (VEGF) expression. Flavonoids contribute to vascular health by reducing metalloproteinases (MMP) and decreasing endothelial activation, exemplified by the downregulation of intercellular adhesion molecule 1 (ICAM-1) and vascular cell adhesion molecule 1 (VCAM-1) expression. Additionally, calcium dobesilate inhibits the effects of serotonin, bradykinin, and histamine on capillary permeability. It also induces the synthesis and release of nitric oxide (NO), fostering vessel relaxation and preventing endothelial cell injury [86] (Figure 7).

Overall, calcium dobesilate, vitamin C, and rutoside are generally well tolerated with no significant side effects reported. Consequently, these medications, particularly when used in combination therapy, merit consideration for the management of PPD.

### 4.5. Immunomodulatory and Immunosuppressant Drugs

There are anecdotal reports of the use of immunomodulatory and steroid-sparing immunosuppressant drugs in PPD. Geller presented a case involving a young woman with Schamberg disease who did not show improvement with oral steroids and pentoxifylline. Subsequently, she was prescribed dapsone at 50 mg per day, resulting in a reduction in the number of skin lesions. Following partial improvement, colchicine at 0.5 mg twice a day was administered, leading to a rapid and nearly complete clearance of skin lesions within just one week [44]. Another instance of colchicine use in Schamberg disease was reported by Cavalcate, involving a woman who did not respond to topical steroid and ascorbic acid treatment. She exhibited an excellent response to colchicine at 0.5 mg per day, and after four months, the treatment was discontinued, with sustained remission observed for the entire 10-month observation period [47]. Colchicine also demonstrated success in treating purpura annularis telangiectodes of Majocchi [91]. In 1995, Tamaki et al. explored the immunomodulatory effects of the antifungal drug griseofulvin in six patients with PPD (five with SD and one with purpura of Doucas and Kapetanakis), observing significant improvement within 1 to 2 weeks [92]. A single study reported the use of aminaphtone in PPD [41]. A young female with SD received 75 mg of aminaphtone twice daily for one month, resulting in the disappearance of purpuric lesions within approximately one week. Notably, there was no relapse of purpura observed one year after discontinuing the medication [41]. The observed effect is believed to be attributed to the immunomodulatory and venoprotective properties of aminaphtone [93]. However, the subsequent efficacy of griseofulvin and aminapthone in PPD has not been investigated.

In 1999, a Japanese team utilized oral tranilast (300 mg/day) in conjunction with topical steroids for treating Schamberg disease in a teenage boy [94]. They observed improvement after one month, with complete resolution of skin lesions achieved within a year [94]. Tranilast primarily functions by suppressing mast cell degranulation, but it may also play an immunomodulatory and anti-inflammatory role by influencing the expression patterns of cytokines and chemokines and modulating inflammasomes. Its impact on the NLRP3 inflammasome has been studied during the COVID-19 pandemic, and the drug has been considered a potential treatment for patients with acute COVID-19 infection [95].

Limited data are available on the efficacy of steroid-sparing immunosuppressants. Methotrexate at a dosage of 15 mg per week demonstrated success in treating purpura annularis telangiectodes of Majocchi, with complete clearance of skin lesions observed after only 4 weeks of treatment [96]. However, recurrence occurred upon discontinuation of methotrexate, but subsequent retreatment led to clearance [96]. In the case of cyclosporin A, an initial dose of 1.5 mg/kg/day, later reduced to 0.74 mg/kg/day due to hypertension, resulted in clinical improvement, as evidenced by a repeated skin biopsy showing a reduction in the lymphocyte infiltration [97].

The effectiveness of these therapies supports an immunologic etiology for PPD. Due to potential side effects, the use of these drugs should be reserved for patients with highly symptomatic disease that is refractory to other treatments.

### 4.6. Energy-Based Devices

Another promising option in PPD treatment is laser therapy. In particular, 595-vascular laser proved to be effective in Schamberg disease [98] and lichen aureus [99,100], and the lesions improved after 3–5 treatment sessions. Non-ablative 1540 nm erbium was used in three men suffering from SD and resulted in 75% clinical improvement after four monthly sessions [101]. The implementation of intense pulsed light (IPL) was described recently by the team of Demidion et al. in a 31-year-old female suffering from SD [48]. Remission was observed after only two sessions and lasted for three years [48].

Photodynamic therapy (PDT) was explored in two females exhibiting a localized variant of PPD, each presenting with a single patch on their lower legs (under 5 cm diameter) [102]. One patient underwent treatment with 5-aminolevulinic acid (ALA) in combination with intense pulse light (IPL), experiencing improvement after three sessions, although the treatment had to be halted due to planned surgery. The other patient received seven sessions of PDT using red light from a Waldman PDT 1200 lamp, with a light dose of 15 J/cm^2^ and a fluence rate of 50 mW/cm^2^. Clinically, the lesion showed improvement, and the histopathological report upon reassessment revealed a decrease in immune cell infiltrate, vessel count, and erythrocyte extravasation [102].

The precise workings of lasers in PPD are yet to be completely understood. Nevertheless, potential explanations of 1540 nm effect include the elimination of hemosiderin deposits and collagen remodeling [101]. The action of 595 nm and IPL might be associated with the wavelength’s preference for oxyhemoglobin and energy delivery to capillaries and intraluminal blood [99]. This, combined with its anti-inflammatory properties, positions laser therapy as a promising choice for localized lesions resistant to topical treatments.

### 4.7. Watchful Waiting Approach

There are currently no established guidelines for the treatment of PPD, and the efficacy of available methods is often limited. Given that PPD is frequently asymptomatic and tends to have a self-limited nature in many cases, adopting close monitoring of the condition without immediately initiating active treatment could be considered. A study by Coulombe et al. focused on seven pediatric patients with various PPD types, including Schamberg disease, lichen aureus, and eczematoid-like purpura of Doucas and Kapetanakis, where no active therapy was pursued. Spontaneous improvement was noted in five out of seven cases [2]. Similarly, Torello et al. described a pediatric group of 13 patients with Schamberg disease who did not receive any treatment. In four cases, the lesions faded within 1 to 4 years, one case showed improvement after 6 years, and eight patients experienced persistence of the disease for 1 to 7 years [1]. A comparative study involving child patients with various PPD types, the majority being lichen aureus and SD, revealed that remission occurred in 42% of those treated with vitamin C and/or rutoside, while 58% of the untreated group showed spontaneous remission [103].

While spontaneous remissions are frequent in Schamberg disease and other pigmented purpuric dermatoses, opting for a conservative approach may be deemed unacceptable by certain patients, considering the physical and psychological impact associated with these chronic conditions. Therefore, a thorough assessment is essential to determine in which patients specific treatment modalities offer advantages over a watch-and-wait strategy.

## 5. Summary and Conclusions

In conclusion, the management of Schamberg disease poses a therapeutic challenge, due to the restricted efficacy of existing interventions, emphasizing the necessity for a combined therapeutic approach. Topical corticosteroids, commonly employed, yield unsatisfactory results, and the recurrence rate upon cessation of treatment remains a concern. Phototherapy, particularly in children, emerges as a viable option, demonstrating good tolerance and therapeutic outcomes, especially in extensive or unresponsive cases. Pentoxifylline, with its multifaceted mechanisms, has shown promise, albeit with higher dosage regimens of 1.2 g a day. The immunomodulatory and immunosuppressant agents, including colchicine, dapsone, methotrexate, and ciclosporin, present encouraging results but should be reserved for highly symptomatic, refractory cases due to potential side effects. The role of hemorheological agents, such as vitamin C, calcium dobesilate, and flavonoids, remains noteworthy, providing an alternative avenue for consideration. Watchful waiting might be an acceptable approach in asymptomatic cases, acknowledging the self-limiting nature of the disease.

Given the various subtypes and multiple factors potentially involved in the pathogenesis and progression of Schamberg disease, a comprehensive understanding of the disease’s natural course is imperative. Future research should focus on standardizing treatment protocols, exploring novel therapeutic avenues, and elucidating the intricate factors contributing to the PPD manifestation and progression. Overall, a tailored, patient-centric approach is essential, considering the diverse clinical presentations and patient preferences in managing these chronic and often perplexing cutaneous disorders. In Table 2, a summary of treatment modalities is provided (Table 2).

## 6. Limitations

While this review aims to provide a comprehensive overview of the current literature on pigmented purpuric dermatoses, it is essential to acknowledge certain limitations that may impact our conclusions. Firstly, the quantity of available studies may vary across different aspects of PPD. While efforts were made to include a diverse range of studies, the overall number and heterogeneity of the available literature could potentially influence the generalizability of our findings. Secondly, variations in treatment protocols, including doses and intervention types, exist among the therapeutic agents discussed in this review. The lack of standardization in these protocols may introduce a source of variability that should be considered when interpreting the results. Lastly, potential disparities between the types of interventions, including the number of treated patients and follow-up period, could affect the comparability of outcomes. We encourage future research to address these limitations by conducting more standardized studies and providing detailed information on treatment protocols, thus enhancing the reliability and applicability of the findings in this field.

## Figures and Tables

**Table 1:** Clinical characteristics of pigmented purpuric dermatoses [3,17].

Variant	Age and Gender	Skin Lesions	Symptoms	Course
Schamberg disease	Middle-aged to older men	Coalescing orange-red macules with pinpoint petechiae, called “cayenne pepper” spots	Generally asymptomatic	Persistent and characterized by frequent relapses and remissions over time.
Pigmented purpuric lichenoid dermatosis of Gougerot and Blum	Elderly men	Purpuric lichenoid papules that merge into plaques	Pruritus	Chronic
Lichen aureus	Children and young adults, mostly men	Solitary golden-orangish papules or plaques Sometimes segmental, along the lines of Blaschko	Generally asymptomatic, but might be itchy	Chronic
Majocchi disease	Children and young adults, mostly women	Red annular macules with central fading and telangiectatic puncta at the periphery	Generally asymptomatic, sometimes itchy or with burning sensation	Chronic, often follows a recurrent course
Eczematoid purpura of Doucas and Kapetanakis	Adult men	similar to Schamberg disease, but with scaling	Intense itch	Rapid onset and chronic course

**Table 2:** A summary of treatment strategies for pigmented purpuric dermatoses.

Drug	Recommended Regimen	Remarks
Oral corticosteroids [44,45]	-	Might be effective, but should be avoided due to a high rate of recurrencies after dose tapering.
Topical corticosteroids [2,36,39,40,41,43]	Medium to high potency, once a day	Might be effective, but often has recurrencies. To be considered in children, limited and itchy lesions. Be cautious of side effects related to a prolonged use.
Topical calcineurin inhibitors [2,51,52]	-	Might be effective. Better side effect profile than corticosteroids.
Pentoxifylline [43,44,53,54,55,56,57,58]	400 mg three times a day up to 8 weeks	Relatively high efficacy. Fewer side effects compared to other treatments.
UVB-NB [2,37,46,49,76,77,78,79]	3 times a week for about two months	Relatively high efficacy. Maintanance therapy of twice weekly for 3 weeks and once weekly for another 3 weeks might be necessary for long-term remission. Retreatment usually successful. Recommended especially in extensive skin lesions and in children.
PUVA [37,42,70,71]	Up to 30 sessions	Relatively high efficacy. Recommended especially in extensive skin lesions.
Vitamin C and rutoside [2,45,84]	500 mg of vitamin C and 50 mg of rutoside two times a day	Partial remission reported in a few cases.
Rheological drugs combinations	diosmin/hesperidin/Euphorbia prostata extract/calcium dobesilate [86], antihistaminic drug/topical steroid/ascorbic acid/rutoside/hesperidin/diosmin [49], vitamin C and calcium pantothenate [85]	Might be effective, but no standarized drug combinations and dosages.
Dapson [44]	50 mg one time a day	One case report with a partial response.
Colchicine [44,47,91]	0.5 mg two times a day	High efficacy reported in a few patients.
IPL, PDT, Lasers [48,98,99,100,101,102]	-	High efficacy in localized lesions.
Methotrexate, cyclosporin A [96,97]	-	Should be reserved for highly symptomatic patients with failure of other treatments.

## Data Availability

The data that support the findings of this systematic review are available from the following repositories: SCOPUS (https://www.scopus.com) on 21 December 2023 and PUBMED (https://pubmed.ncbi.nlm.nih.gov/) on 21 December 2023. All relevant articles included in this review can be accessed through these databases.
